# Flemingia philippinensis Flavonoids Relieve Bone Erosion and Inflammatory Mediators in CIA Mice by Downregulating NF-*κ*B and MAPK Pathways

**DOI:** 10.1155/2019/5790291

**Published:** 2019-02-17

**Authors:** Guangchen Sun, Congcong Xing, Luting Zeng, Yijie Huang, Xin Sun, Yingqin Liu

**Affiliations:** ^1^Pharmacy College, Guilin Medical University, Guilin, Guangxi, China; ^2^Medical College, Xiamen University, Xiamen, Fujian, China; ^3^Biotechnology College, Guilin Medical University, Guilin. Guangxi, China

## Abstract

**Background:**

The dry root of Flemingia philippinensis has been widely used in the treatment of rheumatism, arthropathy, and osteoporosis in traditional Chinese medicine; the therapeutic effects of Flemingia philippinensis are associated with antiarthritis in traditional Chinese medicine theory. This study was undertaken to investigate the mechanism of bone erosion protection and anti-inflammatory effect of Flemingia philippinensis flavonoids (FPF) in collagen-induced arthritis (CIA) in mice.

**Methods:**

Flavonoids were extracted from the dry root of Flemingia philippinensis. Collagen-induced arthritis in C57BL/6 mice was used as a rheumatoid arthritis model, and the mice were orally fed with FPF prior to induction to mimic clinical prophylactic therapy for a total of 39 days. After treatment, histology and immunohistochemistry staining were performed, and the levels of anti-collagen type II (CII) antibody and inflammatory mediators, as well as the key proteins of nuclear factor kappa-B (NF-*κ*B) and mitogen-activated protein kinase (MAPK) pathways, were detected in the samples taken from ankle joints, plasma, and paws.

**Results:**

FPF administration significantly suppressed the paw swelling and arthritic score in CIA mice. FPF reduced inflammatory infiltration and pannus formation, articular cartilage destruction and osteoclast infiltration, and the expression of MMP-9 and cathepsin K in the ankle joint. FPF inhibited plasma anti-CII antibody levels and the production of inflammatory cytokines and chemokines in CIA paws. FPF treatment suppressed the activation of NF-*κ*B as indicated by downregulating the phosphorylation of NF-*κ*B p65 and mitogen-activated protein kinases in CIA paws. Additionally, FPF significantly inhibited inflammation signaling by suppressing the activation of activator protein-1 subset and signal transducers and activators of transcription 3 (STAT3).

**Conclusions:**

Our data suggest that FPF might be an active therapeutic agent for rheumatoid arthritis and the preventive effect of FPF on arthritis is attributable to an anti-inflammatory effect on CIA by preventing bone destruction, regulating inflammatory mediators, and suppressing NF-*κ*B and MAPK signaling pathways.

## 1. Introduction

Flemingia philippinensis (Merr. et Rolfe) is a shrubby herb that belongs to a family of Leguminosae; its roots are popularly used to treat rheumatism, arthropathy, leucorrhea, menalgia, menopausal syndrome, chronic nephritis, and osteoporosis in traditional Chinese medicine [1]. Previous studies showed that the roots of Flemingia philippinensis contain a variety of flavonoids, especially prenylated isoflavones, which showed anti-inflammation, antiestrogen, antitumor, cytotoxic, immunosuppressive, and antioxidant activities [1–7]; however, its role on rheumatoid arthritis needs to be further studied.

Rheumatoid arthritis (RA) is a chronic autoimmune disease characterized by synovial hyperplasia, inflammatory cell infiltration, pannus formation, and cartilage and subchondral bone erosion. Continuous synovium inflammation subsequently results in severe deformity and loss of function. Studies have shown that many cells are involved in the pathogenesis of RA, including monocytes/macrophages, T cells, and B cells. Inflammatory cells infiltrate the synovium and further activate the release of cytokines, chemokines, autoantibodies, and matrix metalloproteinases (MMPs) [8]. Furthermore, a wide range of proinflammatory cytokines including interleukin- (IL-) 1, IL-6, IL-12, or IL-23, tumor necrosis factor- (TNF-) *α*, and chemokines such as monocyte chemoattractant protein- (MCP-) 1 coordinate with M-CSF/RANK signals to promote osteoclast-mediated bone erosion [9–11]. Of particular note, induction of these inflammatory cytokines is mediated by the signal pathways including nuclear factor-*κ*B (NF-*κ*B), mitogen-activated protein kinases (MAPKs), activating protein- (AP-) 1 and signal transducers, and activators of transcription 3 (STAT3). NF-*κ*B is a key transcriptional regulator that plays a central role at the onset of inflammation following I-*κ*B degradation [12, 13]. Previous studies have shown that NF-*κ*B plays a key role in mouse models of arthritis, and blocking NF-*κ*B has a dramatic effect in preventing the disease [14, 15]. The MAPK signaling pathway including extracellular signal-related kinase (ERK), p38, and c-Jun N-terminal kinase (JNK) positively regulates a number of cytokines, chemotactic factors, and other enzymes and is thought to have a key role in the pathogenesis of RA [16, 17]. Therefore, the inhibitors of NF-*κ*B or MAPK may be effective as an antiarthritis agent.

Therefore, this study was conducted to investigate the protective effects of Flemingia philippinensis flavonoids (FPF) on the bone erosion in a CIA mouse model and the expression of cytokines and transcription factors. Our results showed that FPF has therapeutic effects on the CIA disease by regulating NF-*κ*B and the MAPK pathway protein. Furthermore, the results of this study may provide evidence for the future clinical application of FPF in the treatment of RA.

## 2. Materials and Methods

### 2.1. Reagents and Antibodies

The Flemingia philippinensis root was purchased from a traditional herb market in Guilin, China, and authenticated by a taxonomist. The voucher specimen was stored in Pharmacy College, Guilin Medical University, Guilin, China. Chicken type collagen (CII) and Complete Freund's Adjuvant were purchased from Sigma-Aldrich. Antibodies to the following proteins were purchased from Santa Cruz Biotechnology, Santa Cruz, CA: goat anti-mouse IgG2a-HRP (sc-2061), MMP-9 (sc-393859), cathepsin K (sc-48353), MCP-1 (sc-52701), phosphorylated STAT3 (sc-8059), phosphorylated I-*κ*B*α* (sc-8404), and phosphorylated NF-*κ*Bp65 (sc-101753); antibodies anti-Mouse IgG1-HRP, phosphorylated JNK1+JNK2+JNK3 (ab124956), phosphorylated p38 MAPK (ab178867), and phosphorylated Jun D+c-Jun (ab208035) were purchased from Abcam; phosphorylated ERK1/2 antibody (4370) was from Cell Signaling Technology, GAPDH antibody was from PeproTech Biotechnology, and ELISA kits for TNF-*α*, IL-10, IL-12/IL-23 p40 (total), and MCP-1 were from eBioscience.

### 2.2. Total Flavonoid Extraction

Flemingia philippinensis' dry root was pulverized, and the powder of the Flemingia philippinensis' dry root was soaked with 70% ethanol for 2 days (solid to liquid ratio 1 : 14) and refluxed twice (2 h each time), and the mixture was filtered. The final product was evaporated to yield total Flemingia philippinensis flavonoids (FPF 18.38%) and stored at 4°C. The mouse dosage of FPF was transferred from human consumption of Flemingia philippinensis herb. The body surface area normalization method has been prescribed [18, 19].

### 2.3. Animal

The male C57BL/6 mice were obtained from the Animal Experimental Center of Guilin Medical University (experimental animal production license number: SCXK Gui 2013-0001; experimental animal use permit number: SYXK Gui 2013-0001). The mice were kept on a 12/12 h light/dark cycle under specific pathogen-free conditions; experimental studies were strictly in accordance with Guilin Medical University Animal Ethics Committee guidelines for the rational use of animals.

### 2.4. Collagen-Induced Arthritis Preparation

Collagen-induced arthritis (CIA) in mice was performed according to the methodology with minor changes [20]. Chicken collagen type II (CII) was dissolved in 0.1 M glacial acetic acid to a final concentration of 2 mg/ml CII, and the solution was emulsified in an equal volume of Complete Freund's Adjuvant (CFA containing 4 mg/ml mycobacterium tuberculosis) overnight at 4°C. On day 0, the mice in the CIA groups were injected with 100 *μ*l emulsion at the base of the tail by an intradermal route under anesthesia. A booster injection of 50 *μ*l of the same emulsion into the ankle subplantar of both hind limbs was given on day 18. The mice were randomly divided into three groups: normal control group (*n* = 10) without any treatment, vehicle group (*n* = 10), and FPF group (*n* = 9). The mice in the FPF group were intragastrically fed with 40 mg/kg body weight FPF daily from day 7 until the end of the experiment on day 46, while the vehicle group received only water. The thickness of the hind paws was measured using a dial gauge (0.01-10 mm, Peacock Japan), and the clinical score was graded as previously described [21].

### 2.5. Histopathology and Immunohistochemistry

The mouse ankle joints at day 46 were fixed for 24 h in 4% paraformaldehyde, and the joints were decalcified in 15% EDTA at RT (pH adjusted to 7.2 by addition of ammonium hydroxide) for 4 weeks. Serial paraffin sections (5 *μ*m) were stained for hematoxylin and eosin (H&E) staining and toluidine blue and tartrate-resistant acid phosphatase (TRAP) staining to identify osteoclasts. Histopathologic scoring was performed as previously described in detail [21]. For immunohistochemistry, ankle paraffin sections were dewaxed and rehydrated, and endogenous peroxidase activity was eliminated by incubating sections in 3% H_2_O_2_; sections were incubated in 1% goat serum for 60 min and then incubated with the primary antibody of MMP-9 and cathepsin K overnight at 4°C, followed by horseradish peroxidase (HRP) secondary antibody at room temperature for 1 h, and the signal was developed with DAB chromogen according to the manufacturer's specifications. The specificity of the staining was confirmed by using matched isotype control antibodies. All sections were counterstained with hematoxylin. Specific expression markers were examined and photographed using a light microscope, and color densities were quantified using the ImageJ software as described elsewhere [22, 23].

### 2.6. ELISA

Heparinized blood samples were taken from all experimental animals on day 46. Plasma was collected by centrifugating whole blood at 3000 rpm for 10 min and stored at -80°C until use. The levels of anti-CII antibodies in the samples were evaluated by ELISA. In brief, 96 wells were coated overnight at 4°C with 5 *μ*g/ml phosphate solution of chicken CII. The plates were then washed and blocked using 1% bovine serum albumin (BSA). Following the extensive washing, the plasma samples in serial dilutions were added to the plates and incubated overnight at 4°C or 1 h at 37°C. The plates were then washed and incubated with HRP-conjugated anti-mouse immunoglobulin IgG, IgG1, IgG2a, and IgM (BA1075, Boster, China) antibodies. After extensive washing, binding of anti-CII antibodies was revealed by 3,3′,5,5′-tetramethylbenzidine (TMB), and wavelength absorbance was detected under 450 nm.

The mouse paws were lysed with Radio Immunoprecipitation Assay (RIPA, 50 mM Tris, 150 mM NaCl, 1% NP-40, and 0.5% sodium deoxycholate) buffer; the extracts were sonicated for 30 s and centrifuged at 20,000 × g for 15 min. The supernatant was collected for measuring the levels of TNF-*α*, IL-10, IL-12/IL-23 p40 (total), and MCP-1 according to the manufacturer's instruction.

### 2.7. Western Blot Analysis

On day 46, the mouse paws and the splenic tissue were homogenized with ice-cold RIPA buffer containing phosphatase inhibitor and protease inhibitor cocktail (S8820, Sigma-Aldrich Shanghai, China), then sonicated 30 s at 4°C, and centrifugated at 12000 rpm for 30 min; the protein concentration in the supernatant was determined by the Bradford method. The protein extracts were subjected to SDS-PAGE and transferred to polyvinylidene difluoride membranes (PVDF). After being blocked with 5% (*w*/*v*) BSA for 2 h, the membranes were immunoblotted with primary antibodies including MMP-9, cathepsin K, MCP-1, phosphorylated (p-) I-*κ*B*α*, p-NF-*κ*Bp65, p-ERK1/2, p-JNK1+JNK2+JNK3, p-p38, p-Jun D+c-Jun, p-STAT3, and GAPDH on a shaker overnight at 4°C. The membranes were then washed and incubated for 1 h with the secondary antibody. The antibody labelling of the protein bands was detected by the enhanced chemiluminescence (ECL) detection system. The gray values measured with the Image Lab software were normalized against GAPDH.

### 2.8. Statistical Analysis

The statistical analysis of the biological data was performed using the SPSS 18.0 software. One-way ANOVA with Student's *t*-test was performed to test the significant differences. *P* < 0.05 was considered statistically significant.

## 3. Results

### 3.1. FPF Suppressed CIA

**Figure 1:**
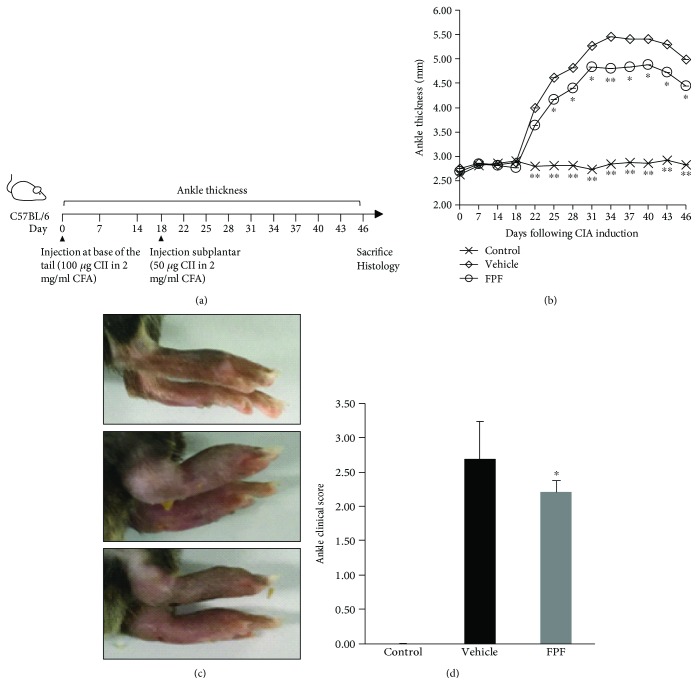
Antiarthritic effect of FPF on arthritis edema and soreness in mice with collagen-induced arthritis. C57BL/6 mice with CIA were treated with 40 mg/kg FPF daily from day 7 to day 46. A schematic diagram of CIA model establishment (a), hind paw thickness (b), representative photographs of the hind paws of CIA mice on day 46 (c), and clinical score on day 46 (d) were shown. Values are the mean 7-8 ± SEM of two independent experiments by one-way analysis of variance (ANOVA). ^∗^*P* < 0.05 and ^∗∗^*P* < 0.01 compared with the vehicle group with CIA.

The induction of collagen-induced arthritis is outlined as in Figure 1(a). The effect of FPF on disease progression in CIA mice was investigated by assessing the development of inflammation. The paw thickness was measured along the experiment, and the clinical disease activity was scored at the end of the experiment. The mice in the control group did not develop paw swelling. The disease activity in vehicle-treated mice with CIA first appeared after subplantar injection on day 18, and the severity of the disease increased gradually in CIA mice. On the 46th day of CII injection, the paw swelling of the vehicle-treated mice increased (5.18 ± 0.36 mm), but the paw swelling was reduced to 4.62 ± 0.41 mm when the mice were given 40 mg/kg FPF (Figure 1(b)).  Figure 1(c) shows typical swelling of the paws of the mouse groups: normal control mice, collagen-induced control mice, and FPF-treated mice, 46 h after CII injection. The photographs clearly show that the paw of the mouse treated with FPF is less swollen than CII-induced vehicle-treated mouse's paw. On day 46, the clinical score of the paws in the vehicle-treated CIA mice reached 2.69 ± 0.55 but was significantly reduced by 2.20 ± 0.17 in the FPF-treated mice (Figure 1(d)).

### 3.2. FPF Attenuated the Histological Parameters of CIA Mice

**Figure 2:**
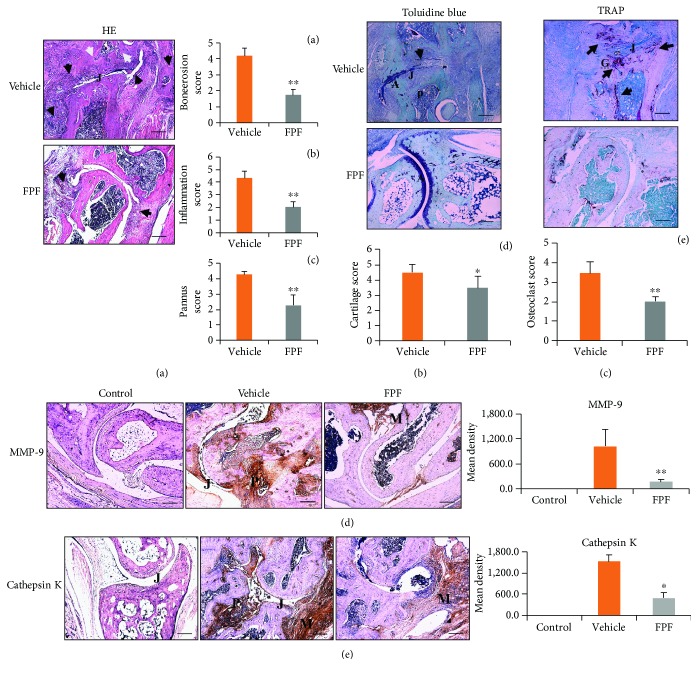
Effect of FPF on inflammation and cartilage loss in CIA mice. Starting at day 7 after the CII challenge, mice in the FPF group were treated daily with 40 mg/kg FPF until day 46; mice in the vehicle group received water alone. H&E staining showing that the vehicle group had inflammatory tissue (black arrows) and erosion (white arrows), but the FPF group only had inflammatory tissue (black arrow) and no erosion (a). Toluidine blue staining shows a decreased amount of proteoglycans and an incomplete staining (black arrows) of the joint in the vehicle group, while the FPF group shows a relatively normal amount of proteoglycans and a complete cartilage (purple) and bones of the joint (b). TRAP staining showing the destruction of cartilage and invading pannus with a massive amount of osteoclast infiltration in the vehicle group; these changes were mitigated in the FPF treatment group (c). Immunohistochemistry for cathepsin K and MMP-9 and the density grades (d, e). Inflammation infiltration, bone erosion, and pannus formation (A, B, C), cartilage destruction (D), and osteoclast activity (E) were graded for each limb and ankle section for H&E staining, toluidine blue staining, and TRAP staining (0–5 per section). The results show the mean of the three mice per group ± SEM. ^∗^*P* < 0.05 and ^∗∗^*P* < 0.01 compared with the vehicle group. A: articular cartilage; J: joint space; M: synovial membrane; P: pannus formation. Magnification: ×50. Bar: 200 *μ*m.

Histology and immunohistochemistry are shown in Figure 2. H&E staining revealed that the FPF-fed group showed limited inflammatory cell infiltration, cartilage erosion, and tissue and pannus formation during CIA joint formation compared with the vehicle group (Figure 2(a), A, B, and C). We qualitatively determined the amount of cartilage proteoglycan by toluidine blue staining, which showed a decreased proteoglycan in the joint and an incomplete staining of the joint in the vehicle group, meaning serious damage occurred in the cartilage. However, a relatively normal proteoglycan with a complete cartilage following FPF treatment implies that FPF can protect the proteoglycan against CIA in mice (Figure 2(b), D). TRAP staining showed that the cartilage was mostly damaged and pannus was formed in the vehicle group, a large number of osteoclasts infiltrated around the bone marrow cavity and pannus, and the injured cartilage and growth plates in the vehicle group had osteoclast distribution. However, these changes were attenuated in the FPF group, indicating that FPF can reduce osteoclast invasion and bone destruction as well as pannus formation (Figures 2(c), E). To clarify whether the cellular components of the inflamed tissue are affected by FPF treatment, the expression of cell-specific markers of the proteinase was quantified by immunohistochemistry. The results suggested that the expressions of MMP-9 and cathepsin K in both the vehicle group and the FPF group were increased compared with those in the control group, but the expressions of both factors in the FPF group were significantly lower than those in the vehicle group, as demonstrated by optical density determination (Figures 2(d) and 2(e)).

### 3.3. FPF Regulated Anti-CII Antibody Levels in Plasma of CIA Mice

**Figure 3:**
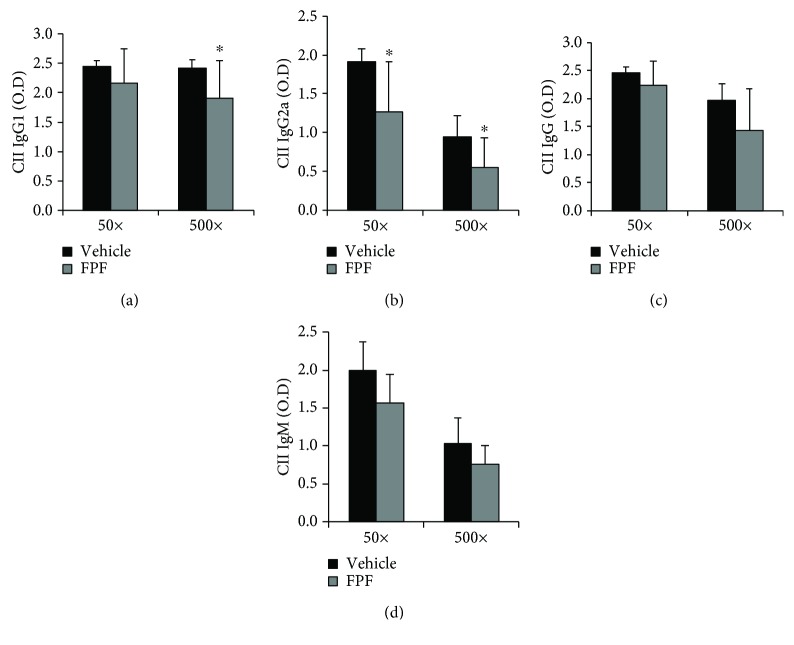
Effect of prophylactic administration of FPF on antibody response to CII in CIA mice. Starting at day 7 after the CII challenge, gavage was performed daily with water and 40 mg/kg FPF until day 46 on the mice. The mice were euthanized, and their plasma was collected for measurement of IgG, IgG1, IgG2a, and IgM against CII. Bar plot ± SEM represent plasma concentrations. ^∗^*P* < 0.05 compared with the vehicle group by Student's *t*-test.

We next examined whether FPF treatment could modulate humoral immune responses by assessing antibody production in CIA mice. The anti-type II collagen production of total IgG, IgM, IgG1, and IgG2a in the plasma and the spleen of CIA mice was determined with different concentrations. A significant downregulation of plasma IgG1 and IgG2a levels in the FPF-treated group was observed compared with the vehicle-treated group (*P* < 0.05) (Figure 3), and the splenic levels of IgG, IgG1, IgG2a, and IgM in the FPF-treated group were significantly reduced when compared with those of the vehicle-treated group (data not shown), indicating that FPF treatment efficiently attenuated the humoral immune response.

### 3.4. FPF Regulated Inflammatory Mediators in CIA Mice

**Figure 4:**
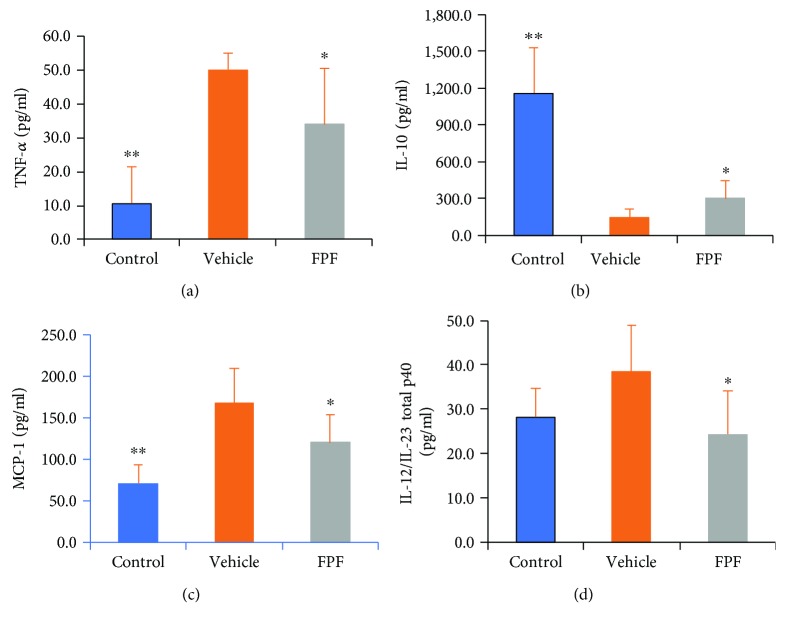
Effect of FPF on proinflammatory and anti-inflammatory mediators in paw tissue. Mice with CIA were untreated or treated with 40 mg/kg FPF daily. On day 46, mice were euthanized, and the cytokines were quantified in paw homogenates by ELISA; the results show the mean of one experiment (seven to eight replicates) ± S.D.^∗^*P* < 0.05; ^∗∗^*P* < 0.01 compared with the vehicle group using Student's *t*-test.

To investigate the effect of FPF on inflammatory mediators for its therapeutic efficiency, the expressions of proinflammatory cytokine (TNF-*α*), anti-inflammatory cytokine (IL-10), and chemokine (MCP-1) were detected by ELISA in mouse paws. The results showed that FPF significantly inhibited TNF-*α* and MCP-1 and significantly upregulated IL-10 expression. In addition, FPF treatment also significantly inhibited IL-12/IL-23 total p40 expression levels (Figure 4).

### 3.5. MAPKs, NF-*κ*B, and STAT3 Pathways Were Involved in Mediating the Effects of FPF on Reducing Inflammation in CIA Mice

**Figure 5:**
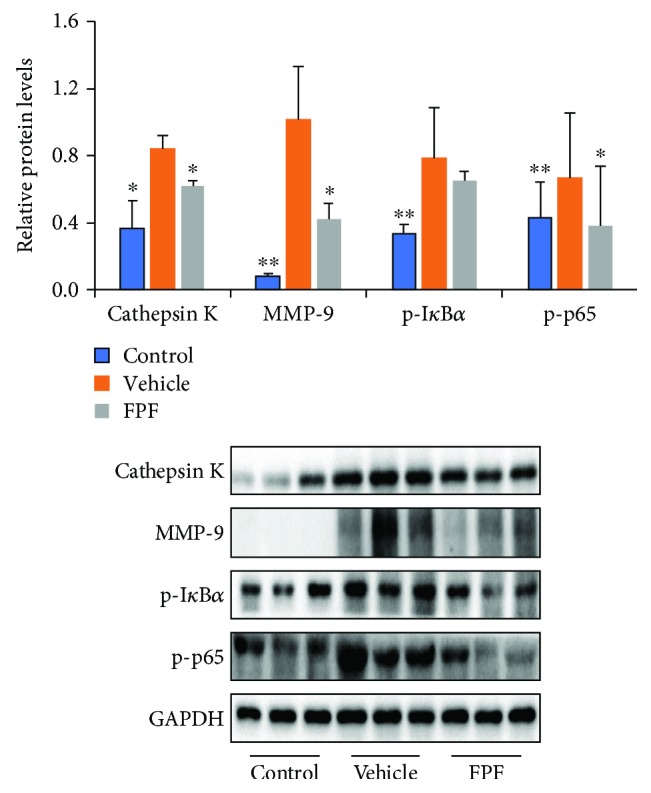
FPF treatment inhibited bone degradation proteinase expression, and nuclear factor kappa-B (NF-*κ*B) activation in the CIA mouse model. C57BL/6 mice were injected intradermally at the base of the tail with CII on day 0 and then challenged with 50 *μ*g CII subplantar at both hind limbs on day 18. On day 46, paws were dissected and total proteins were extracted (*n* = 3-4 each group). Expressions of the indicated cathepsin K, MMP-9, phosphorylated (p-) I-*κ*B*α*, and p-p65 in the paw were examined using western blot and quantified by densitometric analyses. All data are mean ± standard error of the mean (SEM) and ^∗^*P* < 0.05 and ^∗∗^*P* < 0.01 vs. the vehicle group as determined using Student's *t*-test.

**Figure 6:**
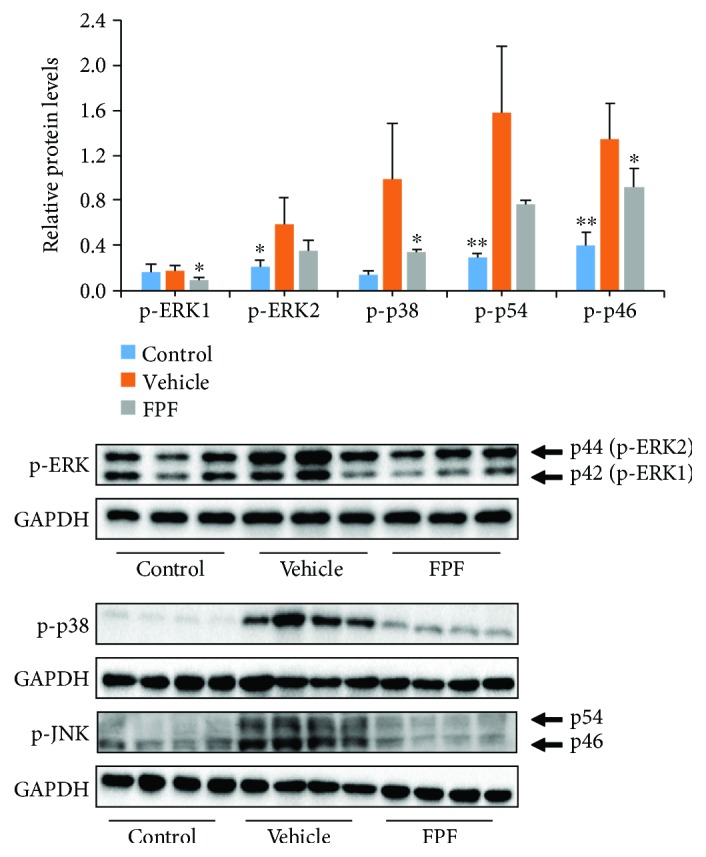
FPF inhibited activation of JNK123, p38-MAPK, and ERK1/2 in the CIA mouse model. C57BL/6 mice were injected intradermally at the base of the tail with CII on day 0 and then challenged with 50 *μ*g CII subplantar at both hind limbs on day 18. On day 46, paws were dissected and total proteins were extracted (*n* = 3-4 each group); expressions of phosphorylated (p-) ERK, p-p38, and p-JNK in the paws were detected using western blot and quantified by densitometric analyses. All data are mean ± standard error of the mean (SEM) and ^∗^*P* < 0.05 and ^∗∗^*P* < 0.01 vs. the vehicle group as determined using Student's *t*-test.

**Figure 7:**
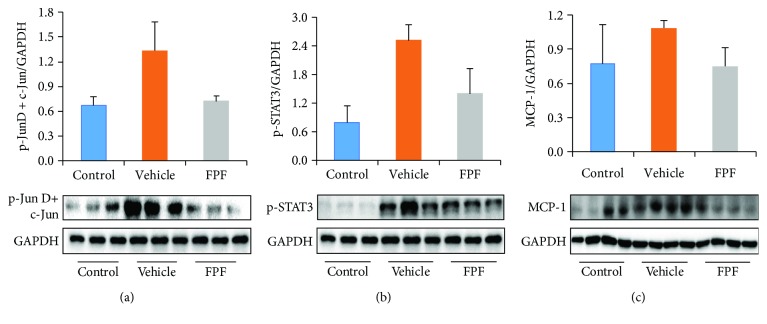
FPF treatment inhibited proinflammatory protein expression and AP-1 and STAT3 activation in the CIA mouse model. C57BL/6 mice were injected intradermally with CII at the base of the tail on day 0 and then challenged with 50 *μ* g CII subplantar at both hind limbs on day 18. On day 46, the paws and the spleen were dissected and total proteins were extracted (*n* = 3-4 each group). Expressions of phosphorylated (p-) Jun D+c-Jun (a) and p-STAT3 (b) in paws and MCP-1 in the spleen (c) were determined using western blot and quantified by densitometric analyses. All data are mean ± standard error of the mean (SEM). ^∗^*P* < 0.05 and ^∗∗^*P* < 0.01 vs. the vehicle-treated control as determined using Student's *t*-test.

Bone and cartilage degradation caused by pannus is associated with increased activity of proteolytic enzymes, including MMPs and cathepsin K. The effects of FPF on the expressions of the two proteolytic enzymes in CIA mice were determined. The results suggested that the expressions of MMP-9 and cathepsin K were significantly inhibited by the treatment of FPF as demonstrated by immunohistochemistry and western blot, respectively (Figures 2(d), 2(e), and 5). In view of the above observations, we further investigated the molecular mechanisms of FPF in CIA mice. NF-*κ*B is an important transcription factor to control the synthesis of a variety of inflammatory cytokines such as TNF-*α*, IL-6, and IL-1, and NF-*κ*B activation requires the phosphorylation of NF-*κ*B [12, 13]. The expression of NF-*κ*B signaling pathway was detected by western blotting analysis. The results revealed that the phosphorylation of p65 was attenuated by FPF administration (Figure 5). MAPKs, including ERK, p38, and JNK, were reported to play key roles in the pathology of RA [24]. We investigated whether MAPK signaling pathways were regulated by flavonoids. To this purpose, we determined the activities of ERK1/2, p38, and JNK in CIA mice. The results demonstrated that FPF administration attenuated the phosphorylation of ERK (p42), p38, and JNK (p46) in paws (Figure 6). AP-1 and NF-*κ*B are key transcription factors that facilitate the expression of various genes involved in immune and inflammatory responses. Therefore, the possible role of AP-1 in the regulation of proinflammatory mediators was investigated, and the result demonstrated that FPF administration attenuated the activities of Jun D/c-Jun in the CIA paws (Figure 7). Apart from MAPKs, STAT3 was also reported to be responsible for inducing the activation of NF-*κ*B [25]. Similarly, we further found that FPF attenuated the activation of STAT3 in CIA mice (Figure 7). Based on the finding that FPF played a role in the regulation of inflammatory mediators in the CIA paws (Figure 3), the expression of MCP-1 in the spleen was detected by western blot. FPF also alleviated the expression of MCP-1 in the spleen (Figure 7). These results clearly demonstrated that FPF inhibited the activation of NF-*κ*B and MAPKs to mediate the production of proinflammatory factors.

## 4. Discussion

Currently, bisphosphonates are the preferred drug for the pharmacological treatment of bone resorption; in contrast, anti-inflammatory ability has disappeared as seen by using the liposomal chlorophosphonate treatment [26]. The most effective anti-inflammatory therapies so far do not alleviate bone loss and joint damage in RA patients [27, 28]. Glucocorticoids used for RA treatment may cause the adverse reactions with bone loss and fracture for long-term use [29]. Natural products, including natural polyphenolic flavonoids, may be beneficial in the development of new drugs for the treatment of autoimmune diseases including RA, due to the lower adverse reactions [30]. The root of Flemingia philippinensis has been traditionally used for treating rheumatism, arthropathy, and chronic nephritis. Studies reported that the extract of the root of Flemingia philippinensis exhibited antioxidative, anti-inflammatory, estrogenic, and antiestrogenic activities. The prenylated flavonoids in this plant acted as bioactive estrogen [1, 31]. Flavonoid compounds in the root of Flemingia philippinensis showed a strong inhibitory activity on lymphocyte proliferation and the low cytotoxicity on splenic lymphocytes stimulated with concanavalin A or lipopolysaccharide, respectively [4]. However, the effects of this plant or its flavonoids on arthritis are poorly investigated. In the present study, our aim was to explore whether FPF acts as a modulator of inflammation and bone destruction in vivo. For this purpose, we for the first time demonstrated the potential therapeutic action of FPF in an experimental model of rheumatoid arthritis, mimicking the clinical situation of prophylactic treatment for RA. FPF had significant protective effects against cartilage erosion and bone destruction in the affected ankle joint, and its protective effect was associated with the downregulation of TNF-*α* and MCP-1, the upregulation of IL-10 in the joints of experimental arthritis animals, and the suppression of MAPK and NF-*κ*B activation in CIA.

The CIA model was characterized by the formation of synovial inflammation, massive bone erosion, and cartilage destruction, ultimately leading to complete loss of the joint architecture. In this study, representative markers for 3 main issues, i.e., synovial inflammation, cartilage erosion, and bone destruction (seen on histology), were attenuated, unraveling the interesting features of FPF in CIA treatment. Proinflammatory factors, such as cytokines TNF-*α*, IL-12, and IL-23 and chemokine MCP-1, play a crucial role in the pathology of synovitis and progressive joint destruction in RA [8, 9, 32–34]. IL-10 is a cytokine with potent anti-inflammatory properties, and it seems to play a dual role in RA by simultaneously suppressing humoral autoimmune response and proinflammatory cytokines [35]. In this study, FPF treatment significantly reduced the levels of TNF-*α*, MCP-1, and p40 subunit of IL-12 and IL-23 in CIA mice and increased the expression of IL-10. It is also well known that an imbalance between pro- and anti-inflammatory cytokine activities is implicated in the induction of autoimmunity, chronic inflammation, and thus joint damage. Proinflammatory cytokines such as TNF-*α* activate the NF-*κ*B pathway, leading to the production of MMPs that in turn mediate tissue destruction [8]. In contrast, anti-inflammatory cytokines such as IL-4 and IL-10 suppress joint damage in RA [36]. The current evidence suggested that FPF shifted the cytokine balance to Th2 cytokines that are beneficial for protecting joint destruction, although FPF treatment attenuated the increase in the levels of both Th1- and Th2-type antibodies IgG2a and IgG1 in CIA mice.

It is well established that NF-*κ*B signaling has been implicated in the progress and development of RA by regulating the transcription of proinflammatory cytokine-, chemokine-, and osteoclastogenesis-related genes including cathepsin K and MMP-9. The products of these genes synergistically enhanced the inflammatory response, which in turn leads to further activation of NF-*κ*B. In fact, persistent NF-*κ*B activation has been found in both animal models and RA patients [14, 15]. Thus, it is clear that NF-*κ*B is an important target molecule for RA therapy. In this study, we found that phosphorylation of p65 was triggered in the paw tissue of CIA mice and that this phosphorylation was suppressed by prophylactic doses of FPF, as demonstrated by the results of western blot for p-p65 and both immunocytochemistry and western blot for cathepsin K and MMP-9.

The transcriptional activity of NF-*κ*B can be further modulated in the nucleus by three MAPK pathways, including p38, ERK, and JNK. MAPK promotes the production of several cytokines such as TNF-*α*, IL-6, IL-12, and IL-23, pannus formation, and cartilage degradation [37–40]. JNK, ERK, and p38 are constitutively expressed in the RA synovial tissue [17, 41] and are liable to be activated in the joint tissue from RA patients [42]. Preclinical experiments with selective inhibitors of p38, ERK, and JNK kinases provide support for their potential targets in human disease [37, 43, 44]. Hence, JNK and p38 inhibition is a potential therapeutic tragedy for the prevention of matrix destruction. Studies have testified that the phosphorylated MAPKs (p38, ERK, and JNK) transfer to the nucleus and initiate the import and accumulation of AP-1 into the nucleus, thereby regulating the target gene expression. An activated JNK or ERK signaling leads to the accumulation of phospho-c-Jun and phospho-Jun D of AP-1 [37, 45]. In this study, FPF treatment reduced the phosphorylation of MAPK signaling pathway, including p38, JNK, and ERK, and further inhibited the AP-1 expression level, suggesting its targeting MAPK signaling pathway. These results suggest that the effects of FPF on the production of inflammatory mediators are likely mediated through the precluding of MAPK pathway in CIA mice.

NF-*κ*B and STAT3 is recognized to be representative transcription factors that play crucial roles in inflammatory signaling. Numerous reports have suggested that both NF-*κ*B and STAT3 collaborate with each other and contribute to inflammation and that this synergistic interaction between the two transcriptional factors may exacerbate inflammatory responses mediated by inflammatory signaling pathways in many diseases, including RA [25]. In this study, we showed that treatment with FPF attenuated the proinflammatory responses, which was accompanied by the simultaneous inhibition of NF-*κ*B and STAT3 in the paw tissues of the CIA mice. Taken together, the anti-RA effects of FPF may be closely related to the simultaneous downregulation of the STAT3 and NF-*κ*B signaling pathways.

## 5. Conclusions

We showed here for the first time that oral FPF administration effectively ameliorated arthritis symptoms in a CIA mouse model including joint inflammation, cartilage damage, and bone destruction, concomitant with inhibiting proinflammatory mediators and increasing anti-inflammatory factors and inhibition of NF-*κ*B and MAPK pathways effectively in vivo. This makes FPF a new type of antirheumatoid drug for further evaluation. We demonstrated here that FPF is effective in inhibiting mouse CIA and reducing associated inflammation. Further investigations, such as time-course analyses using localized cells, can provide more detailed insights into the direct and indirect antiarthritis effects of FPF, and the active compounds would be identified in the future study.

## Data Availability

All the data supporting the findings were shown within the paper and can be requested from the corresponding author.
